# Influence of inclined magnetic field and heat transfer on the peristaltic flow of Rabinowitsch fluid model in an inclined channel

**DOI:** 10.1038/s41598-024-54396-z

**Published:** 2024-02-27

**Authors:** Y. Elmhedy, A. M. Abd-Alla, S. M. Abo-Dahab, F. M. Alharbi, M. A. Abdelhafez

**Affiliations:** 1 Mathematics Department, Faculty of Science, Sohag University, Sohag, Egypt; 2 Mathematics Department, Faculty of Science, South Valley University, Qena, Egypt; 3 Department of Mathematics, Faculty of Science, Umm Al-Qura University, Makkah, Saudi Arabia

**Keywords:** Magnetic field, Heat transfer, Peristaltic flow, An inclined channel, Rabinowitsch fluid model, Biophysics, Mathematics and computing

## Abstract

The recent study is focused on discussion of heat transfer and magnetic field results of peristaltic flow of Rabinowitsch fluid model in an Inclined Channel. In this piece of research, peristalsis’s fundamental problem with heat transfer in the presence of a magnetic field is checked. An incompressible Rabinowitsch fluid is present in an inclined channel, which is considered as the reference for this research. The solutions are devised with the assumptions of long wavelength and low Reynolds number approximations. The resulting equations are then solved exactly by implementing various command of MATHEMATICA subject to relevant boundary conditions. Results are discussed for various flow quantities like temperature, velocity, tangential stress, pressure gradient and rise, and friction force. Computational simulations are performed to determine the flow quantities. This investigation goes beyond mere calculations and examines particle motion to gain deeper insights into flow quantities. Furthermore, this investigates how magnetic field and heat transfer parameters influence these peristaltic flow phenomena. The outcomes of important parameters were plotted and scrutinized. There is amultitude of medical implementations derived from the current consideration, such as the depiction of the gastric juice motion in the small intestine when an endoscope is inserted through it.

## Introduction

Over the past few decades, numerous researchers have become interested in the study of the peristaltic process because of its massive uses in industry and physiology. In physiology, this mechanism occurs in urine movement to bladder through kidney, in various reproductive systems and in the passage of food through the esophagus. Latham^1^ opened the gateway to the study of peristaltic phenomenon. Mishra and Rao^2^ have employed long wave approximation and low Reynolds number to analyze fluid flow under peristalsis in a two-dimensional asymmetric channel in a moving frame of reference. Barton and Raynor^3^ presented study of peristaltic motion in tubes under the assumption of long wave. Furthermore, they also studied the case for low Reynolds number. Fluids with particle suspension have wide application in industrial, chemical and biological processes. In this context, preliminary studies and modeling of two phase problem have been presented by Saffman^4^. Srinivasacharya et al.^5^ analyzed peristaltic propagation of dusty fluid under long wave approximation. Zeeshan et al.^6^ investigated numerically dusty nanofluids with peristalsis in a curved channel. A matter with small pores in it is termed as porous medium. The fluid flows with varying viscosity and density occurs in many industrial processes such as in the reactors with packed beds, in the oil and gas industry. Geological examples include fluid purification, river bed water absorption, motion of oil and water under the ground, limestone, etc. Physiological models of movement through porous passage are.

microblood vessels, a human lung, a gall bladder with stones and a bile duct. Elshehawey et al.^7^ used low Reynolds number and long wavelength to analyze peristaltic propagation of fluid in a permeable medium in an asymmetrical channel. Khan and Tariq^8^ carried out discussion for the second-grade dusty fluid with peristaltic movement through a porous asymmetric passagge with slip. Analysis of MHD dusty fluid with peristalsis in porous medium has been performed by Parthasarathy^9^. Some latest studies made by some other researchers include^10–12^. The study of heat transfer of dusty fluids flow in a channel is an emergent topic due to its application in designing of many industrial and engineering devices. This phenomenon also encounters in fluidization, oil and gas industry, refining crude oil, gas cooling systems, fluid droplet sprays and polymer technology. The study of heat transfer effects on peristaltic pumping of fluids have become the interest of many.

investigators because of its vital role in hemodialysis and oxygenation processes. Lakshminarayana et al.^13^ assumed Reynolds number very small and wave length very large to discuss thermal and slip influence on peristaltic propulsion of conducting Bingham fluid. Iqbal et al.^14^ dealt with peristaltic Sisko fluid propagation in asymmetric channel. Makinde and Gnaneswara^15^ carried out heat transfer analysis of peristaltic Casson fluid flow through permeable passage in asymmetric channel. Ramesh and Devakar^16^ studied heat transfer impacts on MHD second grade fluid under peristalsis flowing through porous medium in asymmetric channel. Hayat et al.^17^ presented impact of thermal radiation on peristaltic flow of dusty fluids. Kalpana and Saleem^18^ presented thermal analysis of dusty fluid flow in an irregular inclined channel under inclined MHD. Vajravelu et al.^19^ studied peristaltic Jeffrey fluid flow with heat transfer with low Reynolds number and long wave approximation. They considered vertical porous passage. Selvi et al.^20^ presented impacts of heat transfer peristaltic propagation of Jeffrey fluid flow by considering inclined porous stratum. Hafez et al.^21^ discussed the properties of second grade fluid with thermal effects travelling through a tube. Zhang et al.^22^ carried out thermal analysis of sinusoidal transport of particle–fluid motion by assuming low Reynolds number and long wave number. Iqbal et al.^23^ conducted the study of peristaltic Maxwell fluid in symmetric passage by considering heat and mass convective conditions. Chandrawat et al.^24^ considered heat transfer influence on unsteady immiscible dusty and non-dusty fluids. Some related articles are^25–28^. In recent years, researchers have extensively focused on the peristaltic flow of Newtonian and non-Newtonian fluids (see for example^31–42^ and several references therein. Some new contributions in heat and mass transfer phenomena have been discussed in Refs.^(43–46)^.

This paper addresses the impact of heat transfer on magnetohydrodynamic (MHD) Rabinowitsch fluid in the context of peristaltic flow. The Rabinowitsch fluid model is crucial in understanding peristalsis, especially considering the fluid's viscosity dependence. This study aims to examine the influence of a magnetic field and heat transfer on the peristaltic transport of a Rabinowitsch fluid, characterized by its variable viscosity, in a two-dimensional symmetric channel. This investigation was conducted under the assumptions of long wavelength and low Reynolds number. Analytical solutions were derived to express velocity, temperature, tangential stress, pressure gradient and rise, and frictional force. These solutions were then utilized to analyze the impact of various novel parameters. The results obtained from this analysis were graphically represented and extensively discussed.

This present work aims to offer a contribution of studying the effects of inclined magnetic field and heat transfer through an inclined channel and Rabinowitsch fluid (particular yield stress effects) on peristaltic phenomena in the cases of pure convection. Main purpose behind performing this study is to develop a mathematical model which can be applied to study peristaltic flow of Rabinowitsch fluid within human body as convective heat transfer. The present study has applications in modelling of peristaltic flow and flow transport through an inclined channel under the effects of peristalsis. The Rabinowitsch fluid model offers an improved understanding of the peristaltic flow and their influence on the efficacy of dr/ug delivery within the human body. This study can also be applied in Chemical Engineering for the mixing and transport of flow.

## Mathematical formulation of the problem

**Figure 1:**
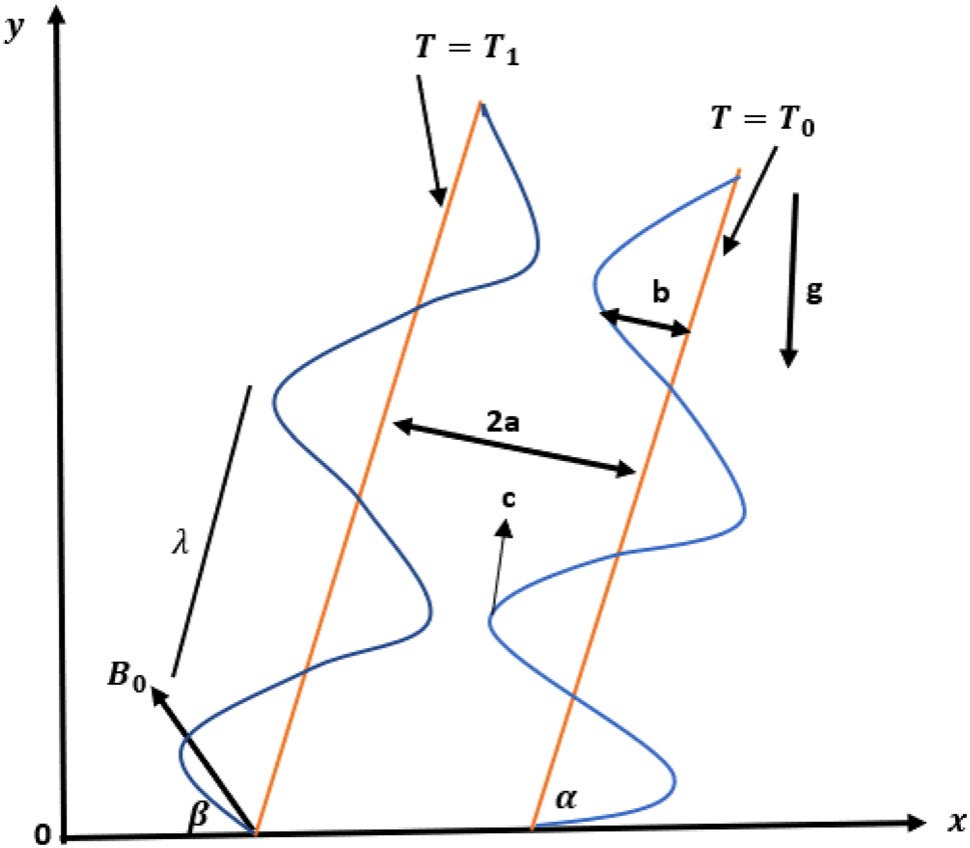
Geometry of the problem.

The paper analyzed an incompressible Rabinowitsch fluid model in peristaltic motion through a uniform and inclined channel (see Fig. 1). The non-Newtonian fluid (Rabinowitsch fluid) filled a two-dimensional symmetric channel of width 2a, and the flow of the fluid, induced by the sinusoidal wave trains of wavelength $\lambda$ and constant speed c, propagated along the channel borders, $b$ represent the wave amplitude and $\overline{t }$ the wave time. Wall surface geometry mathematical expression is defined below ^29^:

1 $$\overline{Y }=\overline{H }\left(\overline{X },\overline{t }\right)=a+b {\text{sin}}\frac{2\pi }{\lambda }\left(\overline{X }-c\overline{t }\right),$$

The governing equations for the flow are ^30^:

2 $$\frac{\partial \overline{{\text{U}}} }{\partial \overline{{\text{X}}} }+\frac{\partial \overline{{\text{V}}} }{\partial \overline{{\text{Y}}} }=0,$$

3 $$\rho \left[\frac{\partial \overline{U} }{\partial \overline{t} }+\overline{U }\frac{\partial \overline{U} }{\partial \overline{X} }+\overline{V }\frac{\partial \overline{U} }{\partial \overline{Y} }\right]=-\frac{\partial \overline{P} }{\partial \overline{X} }+\frac{\partial {\overline{S} }_{\overline{XX}} }{\partial \overline{X} }+\frac{\partial {\overline{S} }_{\overline{XY}} }{\partial \overline{Y} }+\rho g{\text{sin}}\alpha +\rho g{\beta }_{1}\left(T-{T}_{o}\right)-\sigma {B}_{^\circ }^{2}{\text{cos}}\beta \left(\overline{U}\mathrm{cos }\beta -\overline{V}\mathrm{sin }\beta \right),$$

4 $$\rho \left[\frac{\partial \overline{V} }{\partial \overline{t} }+\overline{U }\frac{\partial \overline{V} }{\partial \overline{X} }+\overline{V }\frac{\partial \overline{V} }{\partial \overline{Y} }\right]=-\frac{\partial \overline{P} }{\partial \overline{Y} }+\frac{\partial {\overline{S} }_{\overline{YX}} }{\partial \overline{X} }+\frac{\partial {\overline{S} }_{\overline{YY}} }{\partial \overline{Y} }-\rho g{\text{cos}}\alpha -\sigma {B}_{^\circ }^{2} {\text{cos}}\beta \left(\overline{U}\mathrm{cos }\beta -\overline{V}\mathrm{sin }\beta \right).$$

The heat conduction equation is as follows:

5 $$\uprho {{\text{C}}}_{{\text{p}}}\left[\frac{\partial {\text{T}}}{\partial \overline{{\text{t}}} }+\overline{{\text{U}}}\frac{\partial {\text{T}} }{\partial \overline{{\text{X}}} }+\overline{{\text{V}}}\frac{\partial {\text{T}} }{\partial \overline{{\text{Y}}} }\right]={\text{K}}\left(\frac{{\partial }^{2}T}{\partial {\overline{X} }^{2}}+\frac{{\partial }^{2}T}{\partial {\overline{Y} }^{2}}\right)+{\overline{S} }_{\overline{XX} }\frac{\partial \overline{U} }{\partial \overline{X} }+{Q}_{^\circ }\left(T-{T}_{^\circ }\right).$$

In the laboratory frame $(\overline{X} ,\overline{Y} )$ the flow is unsteady. Hoever, if observed in a coordinate system moving at the wave speed $c$(wave frame) ($\overline{x} ,\,\overline{y} )$ it can be treated as steady. The coordinates and velocities in two frames are

6 $$\overline{x }=\overline{X }-c\overline{t } , \overline{y }=\overline{Y } , \overline{u }=\overline{U }-c\overline{t }, \overline{v }=\stackrel{-}{V,} \overline{p }=\overline{P }$$

where $\stackrel{-}{u,}\overline{v }$ are the velocity components in the wave frame ($\overline{x }$ ,$\overline{y }$ ), $\overline{p }$ is pressures, and $\overline{P }$ is the fixed frame of references.

We introduced the following non-dimensional variables and parameters for the flow.

7 $$\begin{gathered} {\text{x}} = \frac{{\overline{x}}}{\lambda }, y = \frac{{\overline{y}}}{a}, t = \frac{{c\overline{t}}}{\lambda }, p = \frac{{a^{2} \overline{p}}}{c \mu \lambda }, \hfill \\ \delta = \frac{a}{\lambda }, u = \frac{{\overline{u}}}{c}, v = \frac{{\overline{v}}}{c\delta }, Re = \frac{\rho c a}{\mu }, \hfill \\ S_{i j} = \frac{{a\overline{S}_{ij} }}{c\mu }, \theta = \frac{{T - T_{o} }}{{T_{o} }}, \eta = \frac{{Q_{o} a^{2} }}{K}, \hfill \\ B_{r} = \frac{{c^{2} \mu }}{{KT_{o} }}, B_{i} = \frac{ah}{K}, M = B_{o} a\sqrt {\frac{\sigma }{\mu }} . \hfill \\ \end{gathered}$$

where $Br$ is the Brinkman number, $\alpha$ is the angle of inclination of the channel, $\eta$ is the heat source /sink parameter, and $g$ is the gravity field, ${\beta }_{1}$ is the heat transfer coefficie.

Where

$${\overline{S}}_{\overline{X}\overline{X}}=\frac{2\mu }{1+{\lambda }_{1}}\left[1+{\lambda }_{2}\left[\overline{U}\frac{\partial }{\partial \overline{X}}+\overline{V}\frac{\partial }{\partial \overline{Y}}\right]\right]\frac{\partial \overline{U}}{\partial \overline{X}},$$

$${\overline{S}}_{\overline{X}\overline{Y}}=\frac{\mu }{1+{\lambda }_{1}}\left[1+{\lambda }_{2}\left[\overline{U}\frac{\partial }{\partial \overline{X}}+\overline{V}\frac{\partial }{\partial \overline{Y}}\right]\right]\left[\frac{\partial \overline{U}}{\partial \overline{Y}}+\frac{\partial \overline{V}}{\partial \overline{X}}\right],$$

8 $${\overline{S}}_{\overline{Y}\overline{Y}}=\frac{2\mu }{1+{\lambda }_{1}}\left[1+{\lambda }_{2}\left[\overline{U}\frac{\partial }{\partial \overline{X}}+\overline{V}\frac{\partial }{\partial \overline{Y}}\right]\right]\frac{\partial \overline{V}}{\partial \overline{Y}}.$$

## The solution of the problem

According to the above transformations (6) and non-dimensional variables (7), the governing Eqs. (2) - (5) reduce to

9 $$\left( {\frac{c}{\lambda }\frac{\partial u}{{\partial x}} + \frac{c\delta }{a}\frac{\partial v}{{\partial y}}} \right) = 0,$$

10 $$\mathrm{Re \delta }\left[\left({\text{u}}+1\right)\frac{\partial {\text{u}}}{\partial {\text{x}}}+{\text{v}}\frac{\partial {\text{u}}}{\partial {\text{y}}}\right]=-\frac{\partial {\text{p}}}{\partial {\text{x}}}+\updelta \frac{\partial {S}_{xx}}{\partial x}+\frac{\partial {S}_{xy}}{\partial y}+{c}_{1} {\text{sin}}\alpha +{c}_{2} \theta -{M}^{2}{\text{cos}}\beta \left(\left(u+1\right){\text{cos}}\beta -\delta v{\text{sin}}\beta \right) ,$$

11 $$Re \delta \left[\left(u+1\right)\frac{\partial u}{\partial x}+v\frac{\partial v}{\partial y}\right]=-\frac{\partial p}{\partial y}+{\delta }^{2}\frac{\partial {S}_{yx}}{\partial x}+\delta \frac{\partial {S}_{yy}}{\partial y}-\delta {c}_{1} {\text{cos}}\alpha -\delta {M}^{2}{\text{cos}}\beta \left(\left(u+1\right){\text{cos}}\beta -v\delta {\text{sin}}\beta \right),$$

12 $$Re\updelta \left[\left(u+1\right)\frac{\partial \theta }{\partial x}+v\frac{\partial \theta }{\partial y}\right]=\frac{1}{Br}\left[{\delta }^{2}\frac{{\partial }^{2}\theta }{\partial {x}^{2}}+\frac{{\partial }^{2}\theta }{\partial {y}^{2}}\right]+\delta {S}_{xx}\frac{\partial u}{\partial x}+\eta Br\theta .$$

where

$${S}_{xx}=\frac{2\delta }{1+{\lambda }_{1}}\left[1+\frac{{\lambda }_{2}\delta c}{d}\left[u\frac{\partial }{\partial x}+v\frac{\partial }{\partial y}\right]\right]\frac{\partial u}{\partial x},$$

$${S}_{xy}=\frac{1}{1+{\lambda }_{1}}\left[1+\frac{{\lambda }_{2}\delta c}{d}\left[u\frac{\partial }{\partial x}+v\frac{\partial }{\partial y}\right]\right]\left[\frac{\partial u}{\partial y}+{\delta }^{2}\frac{\partial v}{\partial x}\right],$$

13 $${S}_{yy}=\frac{2\delta }{1+{\lambda }_{1}}\left[1+\frac{{\lambda }_{2}\delta c}{d}\left[u\frac{\partial }{\partial x}+v\frac{\partial }{\partial y}\right]\right]\frac{\partial v}{\partial y}$$

After some simplification and using the assumption of long wavelength and low Reynolds number, Eqs. (9) to (12) takes the form

14 $$-\frac{\partial {\text{p}}}{\partial {\text{x}}}+\frac{\partial {S}_{xy}}{\partial y}+{c}_{1} {\text{sin}}\alpha +{c}_{2} \theta -{M}^{2}{{\text{cos}}}^{2}\beta \left(u+1\right)=0,$$

15 $$\frac{\partial p}{\partial y}=0,$$

16 $$\frac{{\partial^{2} \theta }}{{\partial y^{2} }} + \eta Br^{2} \theta = 0.$$

The relative boundary conditions in the dimensionless form are given by

17 $$\begin{gathered} u = 0 , \theta = 0 at y = 0 , \hfill \\ u = - 1, \theta = 1 \,at \,\,\,y = h. \hfill \\ \end{gathered}$$

where $$h = 1 + \varepsilon \sin (2\pi x)$$.

The solutions of Eqs. (14) and (16) subject to boundary conditions (17) can be expressed as

18 $$u=-1+\frac{c}{{a}_{1}}+{c}_{3}{e}^{\sqrt{{a}_{1}}y}+{c}_{4}{e}^{-\sqrt{{a}_{1}}y}+\frac{{a}_{2}{\text{csc}}\left(Brh \sqrt{\eta }\right){\text{sin}}\left(Br y\sqrt{\eta }\right)}{{a}_{1}+{B}_{r}^{2} \eta },$$

19 $$\theta ={\text{csc}}(Br h \sqrt{\eta } ){\text{sin}}(Br y \sqrt{\eta } ),$$

20 $$\frac{dp}{dx}={c}_{1}{\text{sin}}\alpha +\frac{{c}_{3}\sqrt{{a}_{1}} {e}^{h\sqrt{{a}_{1}}}+{c}_{4}\sqrt{{a}_{1}} {e}^{-h\sqrt{{a}_{1}}}-{a}_{1}\left(F+h\right) }{h \left(1+{\lambda }_{1}\right)}+\frac{{a}_{2}{a}_{1}}{h{B}_{r}\sqrt{\eta }\left({a}_{1}+{B}_{r}^{2}\eta \right) \left(1+{\lambda }_{1}\right)},$$

21 $$\Delta {p}_{\lambda }={\int }_{0}^{2\pi }\frac{dp}{dx}dx,$$

22 $${F}_{\lambda }={\int }_{0}^{2\pi }-{h}^{2}\frac{dp}{dx}dx.$$

where

$${c}_{1}=\frac{{a}^{2}\rho g}{c \mu } {c}_{2}=\frac{{a}^{2} \rho g {\beta }_{1}{T}_{o}}{\mu } c=\left(1+{\lambda }_{1}\right) {c}_{1}{\text{sin}}\alpha -\left(1+{\lambda }_{1}\right) \frac{dp}{dx}$$

23 $${a}_{1}={M}^{2}\left(1+{\lambda }_{1}\right) {{\text{cos}}}^{2}\beta , {a}_{2}={c}_{2} \left(1+{\lambda }_{1}\right),$$

$${c}_{3}=1-\frac{c}{{a}_{1}}-{c}_{4},$$

$${c}_{4}=\frac{{a}_{2}}{2\left({a}_{1}+{B}_{r}^{2}\eta \right){\text{sinh}}\left(h\sqrt{{a}_{1}}\right)}+\frac{c\left(1-{e}^{h\sqrt{{a}_{1}}}\right)}{2 {a}_{1}{\text{sinh}}\left(h\sqrt{{a}_{1}}\right)}+\frac{{e}^{h\sqrt{{a}_{1}}}}{2{\text{sinh}}\left(h\sqrt{{a}_{1}}\right) }.$$

The relation between the velocity components (*u*, *v*) and the stream function $\Psi$ is given by

24 $$u=\frac{\partial\Psi }{\partial y},v=-\frac{\partial\Psi }{\partial x}.$$

From Eqs. (18) and (24), one can write

25 $$\Psi =\int udy.$$

## Numerical results and discussion

This section provides the behavior of parameters involved in the expressions of axial velocity $u,$ temperature $\theta$, pressure gradient $\frac{dp}{{dx}}$, shear stress $S_{xy}$, pressure rise, and friction force $F_{\lambda }$. In particular, the variations of Hartman number $M,$ channel inclination Brinkman number $Br$ and the heat source/sink parameter $\eta$, channel inclination angle $\alpha$, gravity field $g$, the ratio of relaxation to retardation times ${\lambda }_{1,}$ the aligned magnetic field $\beta$, and viscosity $\mu$ were examined. The considered parameters’ values were $a = 0.5,\,\,b = 0.5,\,\,d = 1.0$ and $c = 0.3$, while other parameters varied over a range and are given in the caption of the figures. Graphs are drawn to analyse the effects of the relevant parameters mentioned above using the MATHEMATICA a programming language.

**Figure 2:**
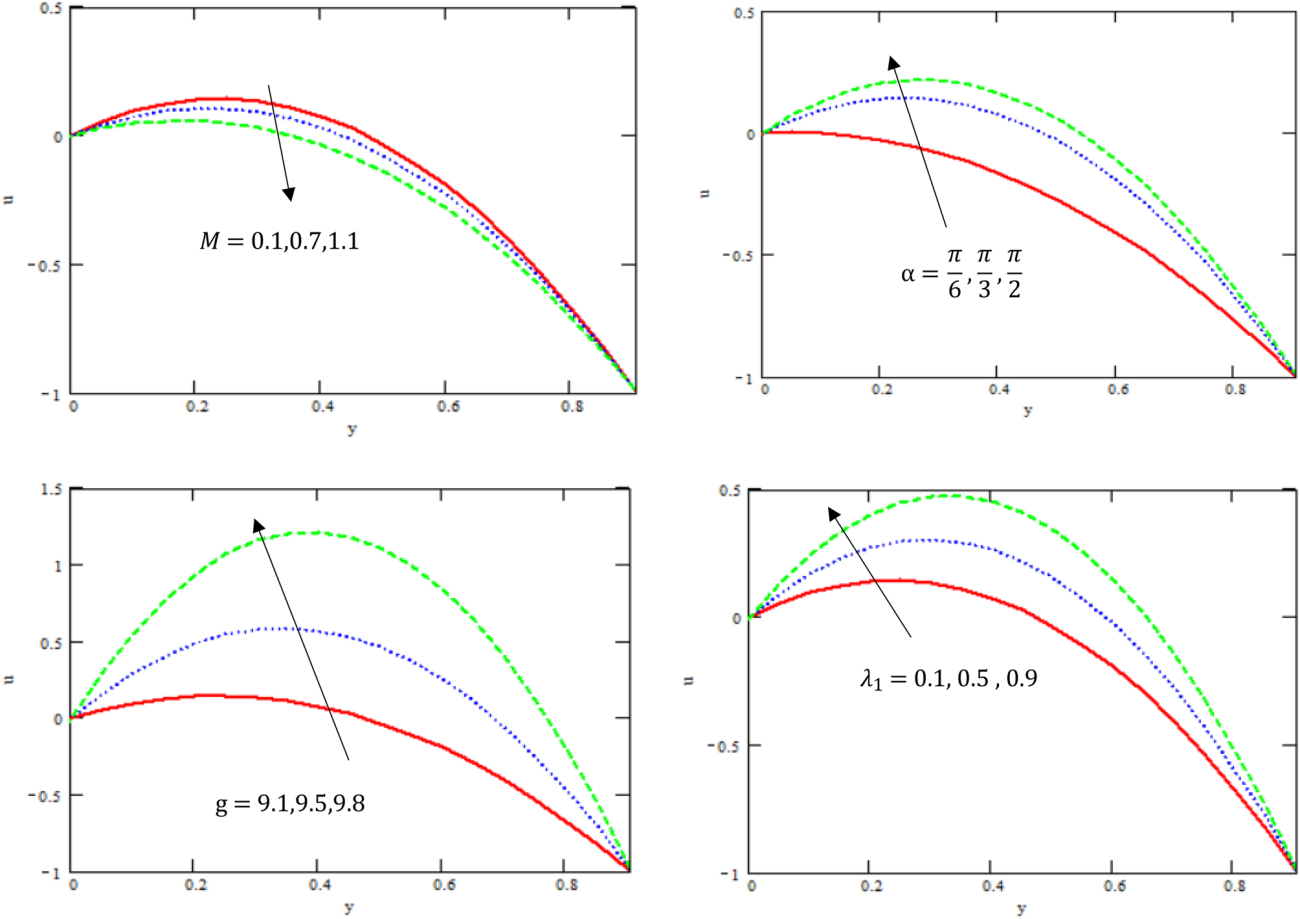
Variation of velocity $u$ concerning the axial-y with different values $g, M,\alpha ,{\lambda }_{1}$.

Figure 2 illustrates the variation of the axial velocity $u$ with respect to $y-axis$ for different values of Hartman number $M$, channel inclination angle $\alpha$, gravity field $g$, and the ratio of relaxation to retardation times ${\lambda }_{1}$. It is observed that the axial velocity decreases with the increase of Hartman number, while it increases with the increase of channel inclination angle, gravity field, and retardation and relaxation times. It is also noted that the velocity satisfied the boundary conditions. Additionally, the impact of the Hartman number is consistent with earlier research by Adnan and Abdul Hadi^29^. The intriguing behavior seen may be due to the intricate characteristics of peristaltic flow, which are well-known for their ability to induce simultaneous increases and decreases^25^ and^26^. One notable observation is that, while the general parabola pattern stays consistent, there are subtle variations in the amplitude of horizontal and vertical particle motion. The observed variability in magnitude serves as a significant indicator that the amplitude of particle motion undergoes alterations when the peristaltic flow through varying depths.

**Figure 3:**
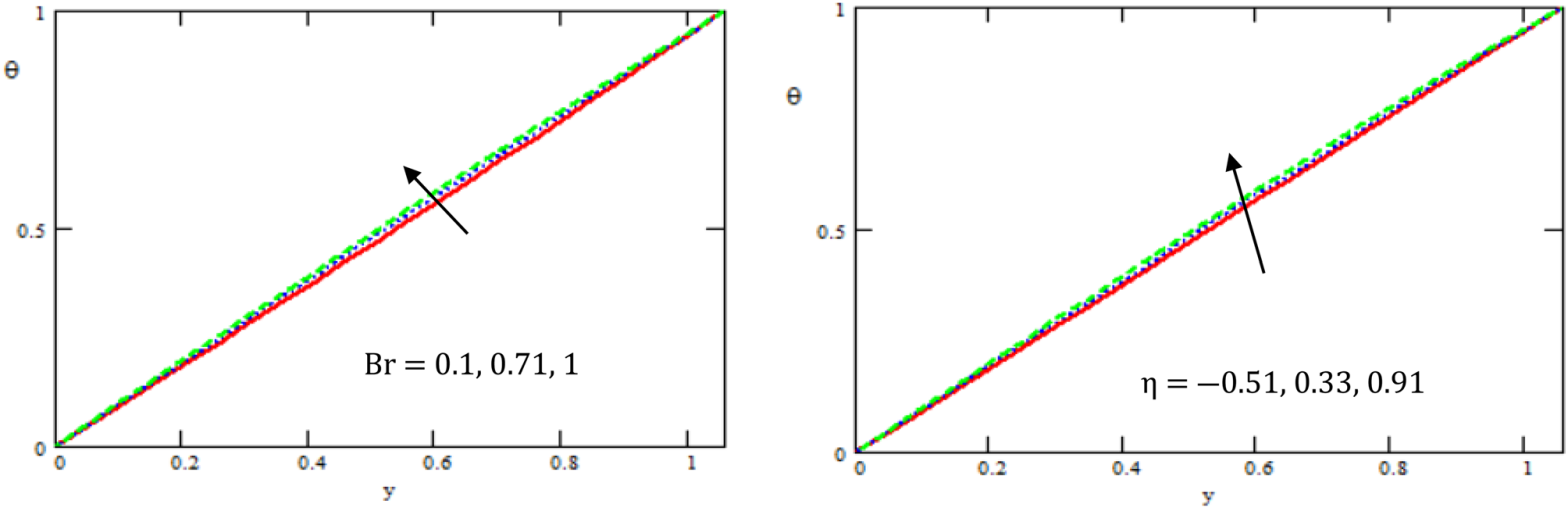
Variation of the temperature $\theta$ concerning the axial-y with different values of $Br, \eta$.

Figure 3 displays the variation of the temperature $\theta$ with respect to $y-axis$ for different values Brinkman number $Br$ and the heat source/sink parameter $\eta$. It is found that the temperature increased with the increase of Brinkman number and the heat source/sink parameter. It is also noticed that the temperature satisfied the boundary conditions. As shown in Fig. 3, the present numerical algorithm agrees with the characteristics- MATHEMATICA solution obtained by Singh et al.^30^. Thus, the present Software MATHEMATICA is appropriate for the subsequent numerical computations in the pure convection case.

**Figure 4:**
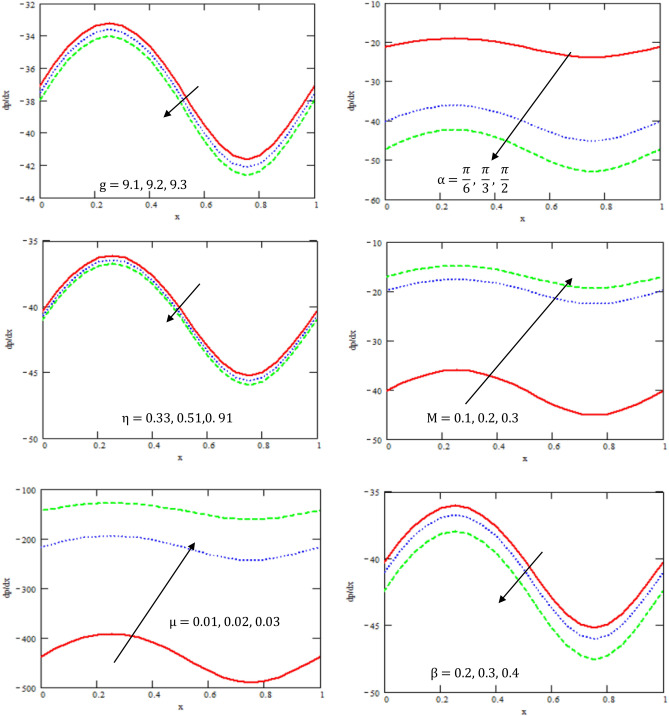
Variation of the gradient of the pressure $\frac{dp}{dx}$ concerning the axial-x with different values of $\beta ,\eta ,\alpha ,\mu ,M,g$.

Figure 4 illustrated the variations of the pressure gradient $\frac{dp}{dx}$ with respect to $x-axis$ for different values of gravity field $g$, channel inclination angle $\alpha$, the aligned magnetic field $\beta$, the heat source/sink parameter $\eta$, Hartman number $M$, and coefficient of viscosity $\mu$. It was found that the pressure gradient decreases with the increase of the gravity field, the inclined angle of the channel, channel inclination angle, magnetic field, and the heat source/sink parameter, while it increases with the increase of Hartman number and coefficient of viscosity. It was found that the pressure gradient has oscillated in the whole range of $x -$ axis. As a result, we can ascertain that the fluid pressure is influenced by the precise strength of the magnetic force. Thus, we can draw the inference that Hartman number of the magnetic force’s intensity empowers us to oversee the fluid pressure. This result is in good agreement with the results obtained by Ramesh and Devakar^16^. From the observations of the results, it has been noted that parameters involved have a similar role in the pressure gradient, since the pressure gradient determines the motion of fluid particles. Fluctuating behavior is shown by pressure gradient attaining its maximum at 0.3, whereas approaching minimum at 0.8 for different values of $\eta ,\,\beta$ and $g$. This shows the existence of high level flow through the channel without the need of greater pressure gradient.

**Figure 5:**
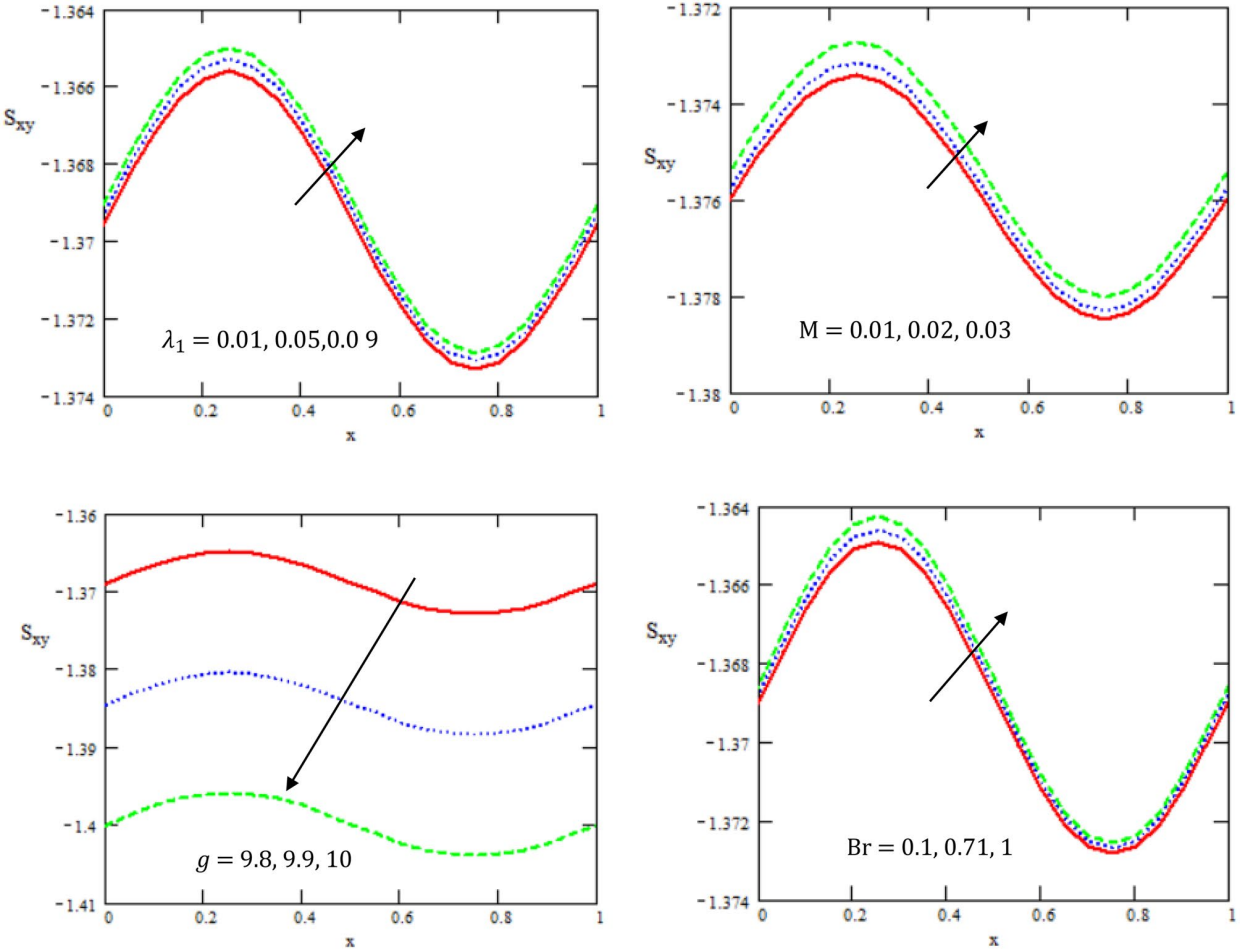
Variations of the shear stress $S_{xy}$ concerning the axial-x with different values of $Br,g,M,{\lambda }_{1}$.

Figure 5 illustrates the variation of shear stress ${S}_{xy}$ with respect to $x-axis$ for different values of the ratio of relaxation to retardation times ${\lambda }_{1}$, Hartman number $M$, Brinkman number $Br$, and gravity field $g$. It is observed that the shear stress increases with the increase of the ratio of relaxation to retardation times, Hartman and Brinkman numbers, while it decreases with the increase of gravity field. The shear stress has oscillated in the whole range of axis $x$.

**Figure 6:**
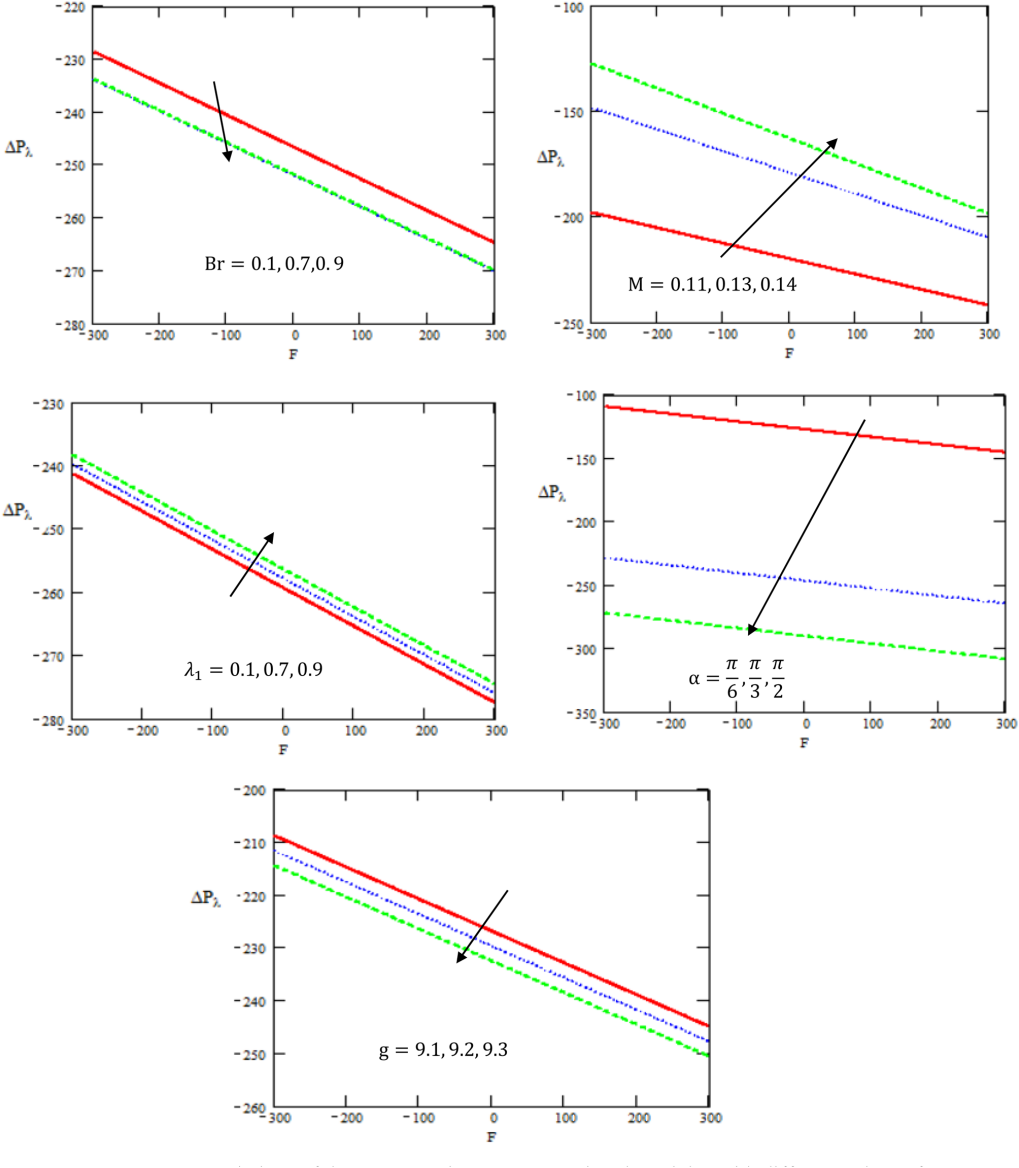
Variations of the pressure rise $\Delta {P}_{\lambda }$ concerning the axial-F with different values of $g,M,\alpha ,{\lambda }_{1},Br$.

Figure 6 depicts the variation of pressure rise $\Delta {p}_{\lambda }$ with respect to the volume flow rate $F$ for different values of Brinkman number $Br$, Hartman number $M$, retardation and relaxation times ${\lambda }_{1}$, the inclined angle of the channel $\alpha$, and gravity field $g$. It is evident that the pressure rise increases with the increase of Hartman number and retardation and relaxation times, while it decreases with the increase of Brinkman number, the inclined angle of the channel, and the gravity field. Peristalsis, which occurred as a result of pressure difference, causes flow rate to be positive in the zone of peristaltic pumping, whereas peristalsis of the tube boundaries produces a free-pumping region. Negative pressure difference helps the peristalsis-related flow in the co-pumping zone.

**Figure 7:**
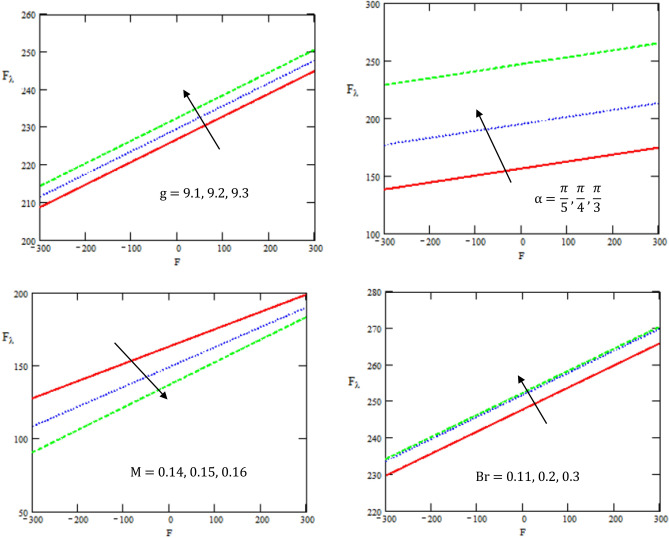
Variations of friction force ${F}_{\lambda }$ with respect to volume flow rate F for values of $M,Br,g,\alpha$.

Figure 7 depicts the variations of the friction force ${F}_{\lambda }$ with respect to volume flow rate $F$ for different values of gravity field $g$, channel inclination angle $\alpha$,Brinkman number $Br$, and Hartman number $M.$ It is observed that the friction force increases with the increase of gravity field, channel inclination angle, and Brinkman number, while it decreases with the increase of Hartman number. It is noticed that the behavior of the friction force is opposite to the behavior of the pressure rise. The frictional force also has the opposite behavior when compared with that pressure rise. This result is similar to that presented in reference^39^.

**Figure 8:**
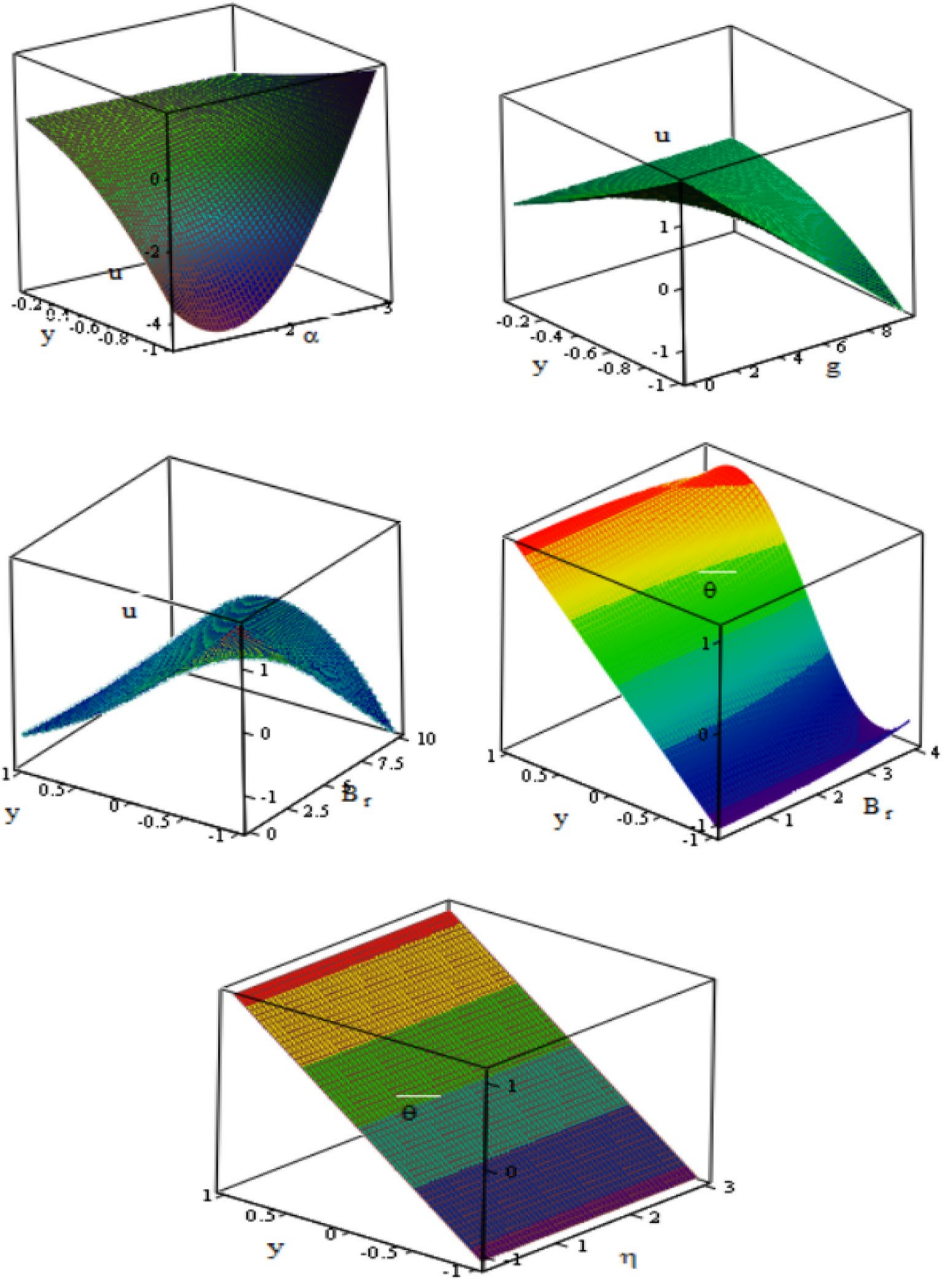
Variations of velocity and temperature in 3-D with respect to $x,\,$ $y-axis$ for different values of $\alpha ,g, {B}_{r},$ $\eta$.

Figure 8 is plotted in $3D$ schematics illustrating the velocity $u$ and temperature $\theta$ with respect to $x$ and $y$ axes in the presence of channel inclination angle $\alpha ,$ gravity field $g$, Brinkman number $Br$, and the heat source/sink parameter $\eta .$ It is observed that the temperature increases with the increase of the Brinkman number and the heat source/sink parameter, while the velocity increases with the increase of channel inclination angle and gravity field. The peristaltic flow in 3D overlap and dampen when $x$ and $y$ increase to reach particle equilibrium. The vertical distance obtained more curves’ significance, as most physical fields are moving in peristaltic flow.

## Streamlines pattern &trapping phenomenon

**Figure 9:**
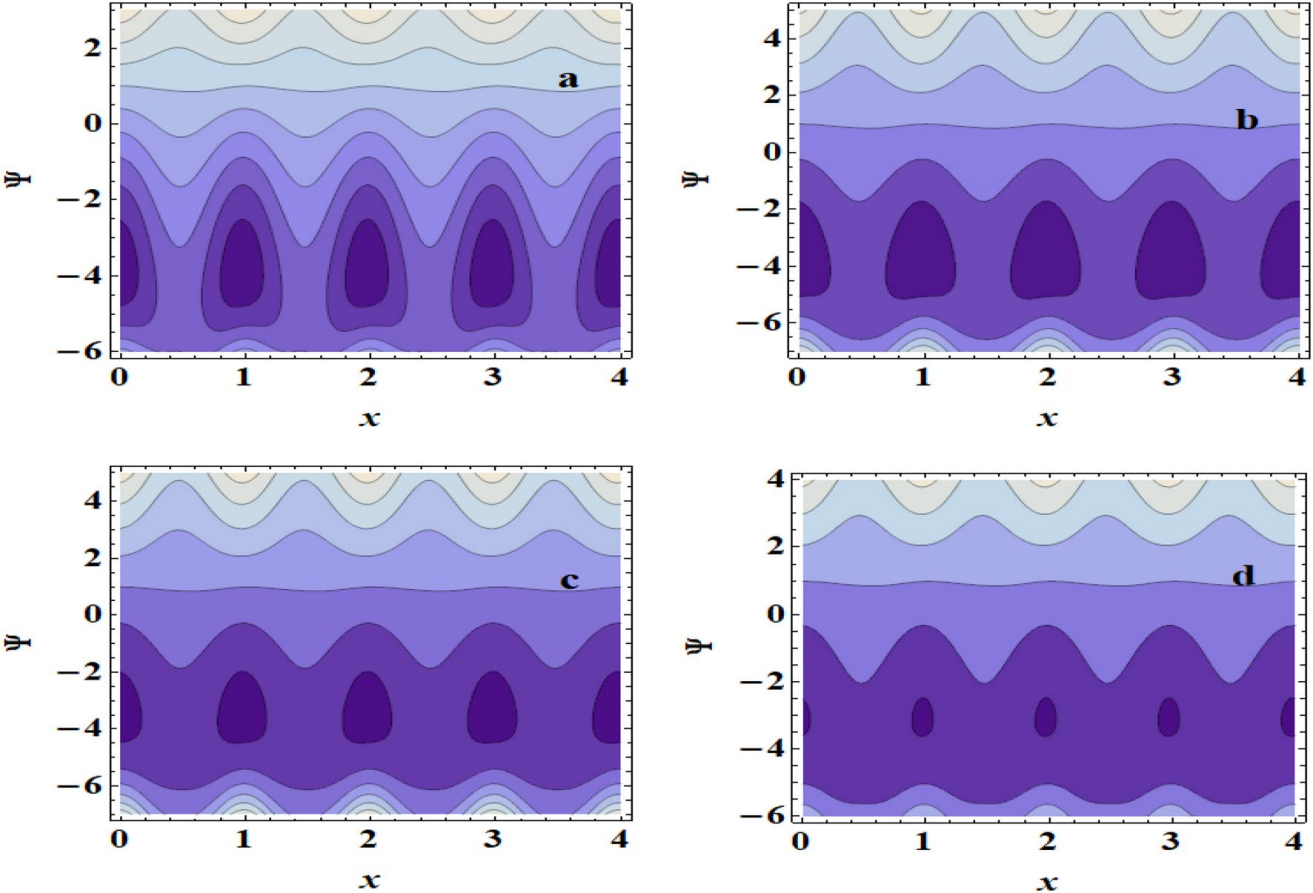
Streamlines for (**a**)$$\beta$$=0, (**b**) $$\beta$$ =0.1, (**c**) $$\beta$$ =0.3, (**d**) $$\beta$$ =0.5.

**Figure 10:**
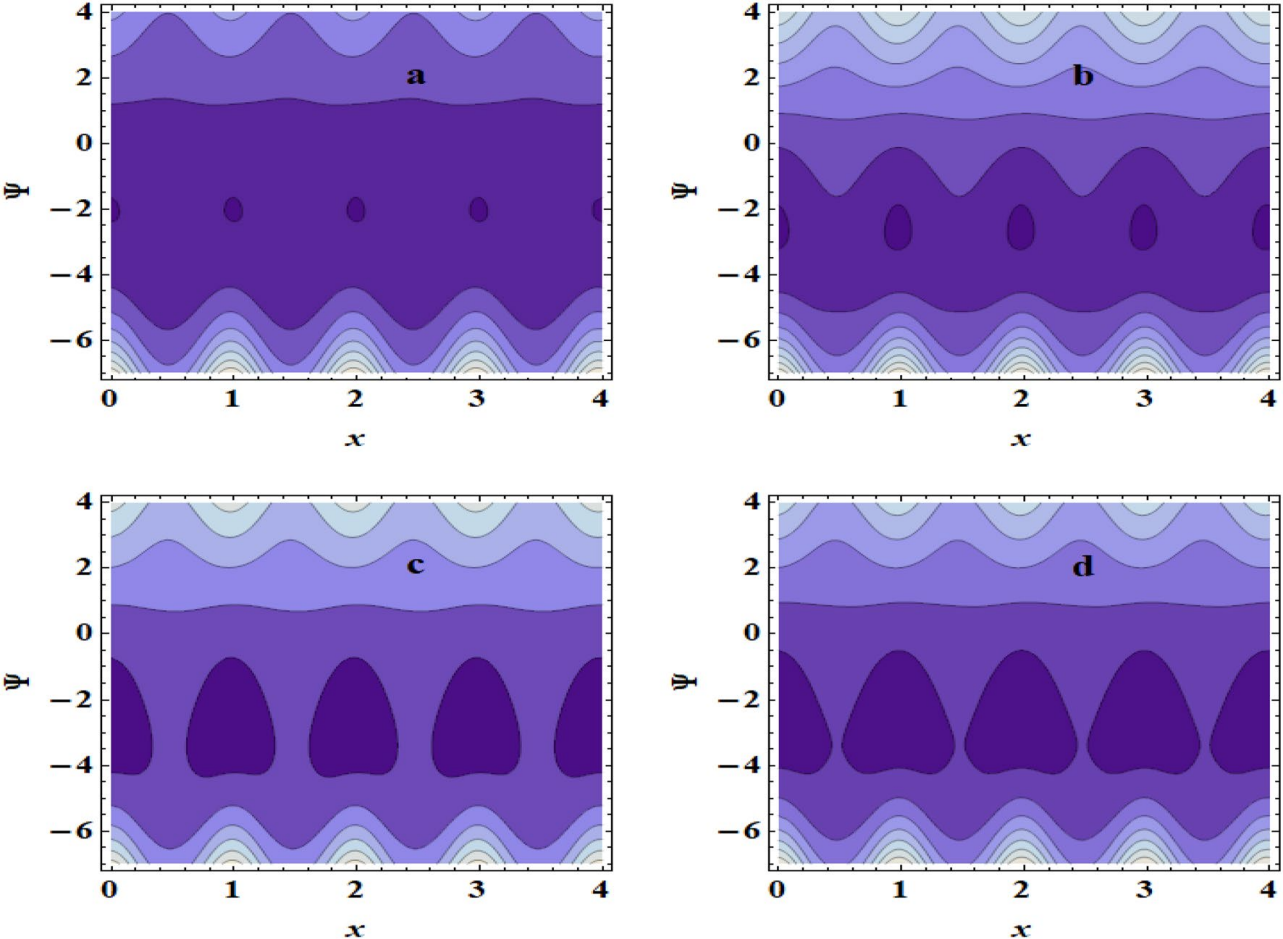
Streamlines for (**a**) $$Br$$=1, (**b**) $$Br$$=3, (**c**) $$Br$$ = 5, (**d**) $$Br$$=7.

**Figure 11:**
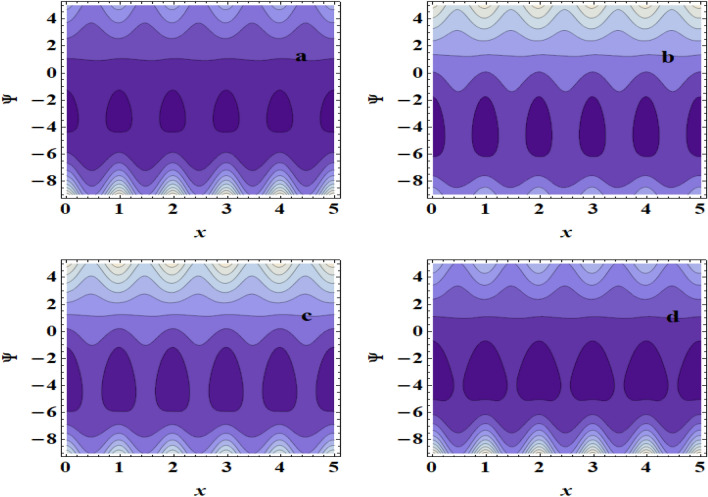
Streamlines for (**a**) $$M$$=0, (**b**) $$M$$=0.1, (**c**) $$M$$=0.5, (**d**)$$M$$=0.8.

The peristaltic mechanism includes a study of the trapping phenomenon. Few streamlines shut during peristalsis, causing the creation of a bolus that circulates inside and advances at the rate of the peristaltic waves. This occurrence is known as trapping. Now, we will discuss this interesting case under the influence of some influences such as the heat source /sink $\beta$, the Brinkman number $Br$ and the Hartman number $M$. Surprisingly, we observe that the trapping phenomenon occurs as shown in Fig. 9a–d, where it is when the value of the heat source /sink $\beta$ increases that the bolus size decreases. In addition to, In Fig. 10a–d, it is seen that the boluses increase in size with increasing $Br$. Also, we found that as $M$ is raised, the trapped bolus's size grows, as shown in Fig. 11a–d. This increase in the size describes the volume of the fluid that is bounded by invariant closed streamlines. Furthermore, compared to the symmetric channel, the size of the trapped bolus is less in the asymmetric channel.

## Conclusion

The most recent research is shown to investigate the impacts of heat transfer and magnetic field on the peristaltic flow of Rabinowitsch fluid. In an inclined channel, fluid flow is taken into consideration through a media. Salient results are as follows.:

1. As the Hartman number grows, axial velocity drops, but it increases as the channel inclination angle, gravity field, and retardation and relaxation time increase.
2. It is noticed that the temperature increases with the increase of Brinkman number and the heat source/sink.
3. This phenomenon causes the temperature to fall duringperistalsis. This indicates that the nature of heat transfer within the system influencestemperature fluctuations.
4. The magnitude of $\frac{dp}{{dx}}$ decreases when there is an increase in the gravity field, the inclined angle of the channel, channel inclination angle, magnetic field, and the heat source/sink parameter.
5. It is observed that frictional force is inversely proportional to pressure rise.
6. The current study, which focuses on magnetoohydrodynamic peristaltic
7. flow, has many applications in chemical engineering.
8. The volume of the trapped bolus increases as the magnetic field and Hatman number increase, while it decreases as the heat source increases.
9. The relationship between the bolus's size and the parameter is straightforward. This shows that variations in the channel geometry and fluid characteristics across the flow pattern affect the bolus's size.
10. The obtained results can be applied to enhance pumping systems in engineering and gastrointestinal functions. This analysis permits body fluids such as blood and lymph to easily move inside the arteries and veins, allowing oxygen supply, waste elimination, and other necessary elements.
11. Researchers in science, medicine, engineering, and fluid mechanics development might find the findings in this paper useful. The results provided here are also expected to serve as similarly good theoretical estimations of numerous prospective fluid mechanical flow governing parameters (heat source /sink $$\beta$$, the Brinkman number $$Br$$ and the Hartman number $$M)$$ connected to peristaltic fluid flow.

## Data Availability

The datasets used and/or analyzed during the current study available from the corresponding author on reasonable request.

## Abbreviations

$\overline{u} ,\,\overline{v}$: The velocity components of the fluid
$a$: Half the thickness of the channel
$$b$$: Wave amplitude
$$\lambda$$: Wavelength
$$\delta$$: Wave number
$$c$$: Constant speed
$$C_{p}$$: Specific heat
$$K$$: Thermal conuctivity
$$B_{r}$$: Brinkman number
$\alpha$: The angle of inclination of the channel
$\beta$: The aligned magnetic field
$$\rho$$: Fluid density
$${\text{Re}}$$: Reynolds number
$F$: Volume flow rate
$\eta$: The heat source /sink parameter
$$g$$: Gravitation acceleration
$$P$$: Pressure
$$\theta$$: Temperature
$\beta_{1}$: Heat transfer coefficient
$$M$$: Hartman number
$\lambda_{1}$: The ratio of relaxation to retardation times
$$F_{\lambda }$$: Friction force
